# Genome-Wide Identification, Expression Profiling, and Evolution of Phosphate Transporter Gene Family in Green Algae

**DOI:** 10.3389/fgene.2020.590947

**Published:** 2020-10-08

**Authors:** Long Wang, Liang Xiao, Haiyan Yang, Guanglei Chen, Houqing Zeng, Hongyu Zhao, Yiyong Zhu

**Affiliations:** ^1^Agricultural Resource and Environment Experiment Teaching Center, College of Resource and Environment Science, Nanjing Agricultural University, Nanjing, China; ^2^Key Laboratory of Plant Nutrition and Fertilizers, Ministry of Agriculture and Rural Affairs, Institute of Agricultural Resources and Regional Planning, Chinese Academy of Agricultural Sciences, Beijing, China; ^3^College of Life and Environmental Sciences, Hangzhou Normal University, Hangzhou, China

**Keywords:** phosphate transporters, phylogenetic analysis, expression pattern, Chlamydomonas, green algae

## Abstract

Phosphorus (P) is an essential nutrient for plant growth and development. Phosphate transporters (PHTs) are *trans*-membrane proteins that mediate the uptake and translocation of phosphate (Pi) in green plants. The PHT family including PHT1, PHT2, PHT3 and PHT4 subfamilies are well-studied in land plants; however, PHT genes in green algae are poorly documented and not comprehensively identified. Here, we analyzed the PHTs in a model green alga *Chlamydomonas reinhardtii* and found 25 putative PHT genes, which can be divided into four subfamilies. The subfamilies of CrPTA, CrPTB, CrPHT3, and CrPHT4 contain four, eleven, one, and nine genes, respectively. The structure, chromosomal distribution, subcellular localization, duplication, phylogenies, and motifs of these genes were systematically analyzed *in silico*. Expression profile analysis showed that CrPHT genes displayed differential expression patterns under P starvation condition. The expression levels of *CrPTA1* and *CrPTA3* were down-regulated, while the expression of most *CrPTB* genes was up-regulated under P starvation, which may be controlled by CrPSR1. The transcript abundance of most *CrPHT3* and *CrPHT4* genes was not significantly affected by P starvation except *CrPHT4-3*, *CrPHT4-4*, and *CrPHT4-6*. Our results provided basic information for understanding the evolution and features of the PHT family in green algae.

## Introduction

Phosphorus (P) is one of the most important macronutrients for algae and land plants. It is a critical component of nucleic acids, phospholipids, and ATP and participates in numerous biochemical pathways, including gene expression, and signal transduction (Lorenzo-Orts et al., 2020). P acquisition is activated by transcriptional induction of genes encoding high- and low-affinity phosphate transporters (PHTs), phosphodiesterases, and phosphatases (Wykoff et al., 1999). In land plants, PHT1 (phosphate transporter 1), PHT2, PHT3, and PHT4 gene families are revealed to encode proteins for uptake and transport of inorganic phosphate (Pi) between the cytoplasm and different subcellular compartments (Wang et al., 2017).

Phosphorus is mainly absorbed as Pi via the proton-coupled H_2_PO_4_^–^ symporters PHT1 gene family, which are located in the plasma membrane of cells at the interface with the external environment in land plants (Nussaume et al., 2011; Wang et al., 2017). The PHT2, PHT3, and PHT4 gene families encode proteins that transport Pi between the cytoplasm and different subcellular compartments in land plants (Guo et al., 2008; Zhu et al., 2012; Liu et al., 2019). PHT2 proteins and some PHT4 members, which locate on chloroplast thylakoid membrane or inner envelope, are responsible for Pi homeostasis in chloroplasts (Guo et al., 2008; Liu et al., 2019). The mitochondrial phosphate transporters (PHT3) play crucial roles in respiration by absorbing Pi into the mitochondrial matrix, where Pi is utilized for the conversion of ADP to ATP (Zhu et al., 2012). A subgroup of conserved MYB transcription factors designated as phosphate starvation response (PHR) and PHR1-like (PHL) have been defined as central regulators of Pi starvation signaling in diverse plants (Sun et al., 2016). Through the physical interactions with a *cis* element, namely, PHR1 binding site (P1BS; GNATATNC), PHR transcription factors are responsible for the transcriptional activation of a considerable proportion of Pi starvation-induced genes, including PHT1 members (Zhou et al., 2008; Bustos et al., 2010; Sun et al., 2016). It has been convinced in paddy rice that OsPHR2 activates *OsPHT1;2* expressions by binding to the P1BS motif present in its promoter region (Wang et al., 2014).

In contrast, in diatoms and green algae, P starvation responses are regulated by an MYB family transcription factor phosphorus starvation response 1 (PSR1), which regulates Pi acquisition through the up-regulation of phosphatases and Pi transporters (Wykoff et al., 1999; Kumar Sharma et al., 2020). Green algae resemble the yeast in containing genes encoding both H^+^/Pi (plant-like, PTA) and the Na^+^/Pi (animal-like, PTB) transporters. Physiological evidence suggests that Na^+^ uptake is necessary for Pi influx in *Chara corallina* under P-deficient conditions (Reid et al., 2000; Mimura et al., 2002). This is related to the sequence conservation and structural similarity of PTB proteins to the metazoan PHT and fungal PHT, which are known to be functional plasma membrane Na^+^/Pi symporters (Kobayashi et al., 2003; Moseley et al., 2006). PHOSPHATE TRANSPORTER A (PTA) genes were homologous to PHO84 in yeast and encode the high-affinity H^+^/Pi cotransporter (Moseley et al., 2009). The function of the PHT gene family has been extensively studied in land plants; however, the function of the PHT gene family in green algae is still largely unknown.

In this study, we identified 25 CrPHT genes in a model green alga, *Chlamydomonas reinhardtii*. We systematically analyzed the gene structures, chromosome localizations, phylogeny, and expression patterns in response to P starvation in both wild-type and *psr1* mutant backgrounds. Our work will provide a foundation for the further analysis of PHT genes function in green algae and the different mechanisms of Pi transport between green algae and land plants.

## Results

### Phylogenetic Relationship of PHTs in Plants

**FIGURE 1:**
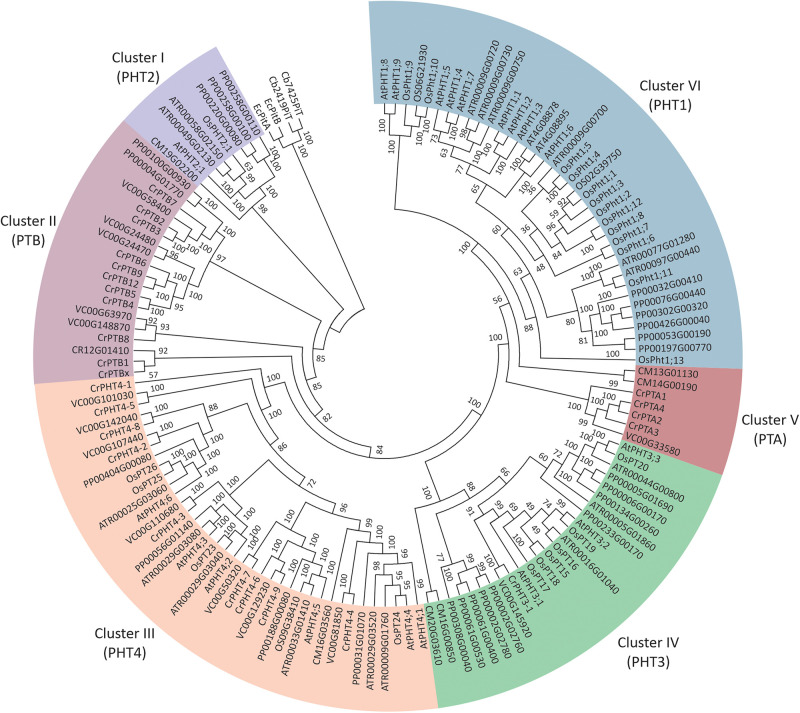
Phylogenetic analyses of phosphate transporter genes in *Arabidopsis thaliana* (At), *Oryza sativa* (Os), *Amborella trichopod*a (Atr), and *Volvox carteri* (Vc). *Physcomitrella patens* (Pp) and *Chlamydomonas reinhardtii* (Cr). Four PHT genes from *Escherichia coli* and Cyanobacteria were selected as outgroup. Multi-sequence alignments were performed by Clustal Omega, and a phylogenetic tree was reconstructed by IQTREE using the maximum likelihood method. WAG + F + R6 was the best-fitting evolutionary model selected by ModelFinder under Bayesian Information Criterion.

To investigate the phylogenetic relationships among PHTs in green plants, a phylogenetic tree was reconstructed by maximum likelihood method using the PHT protein sequences from green plants, bacteria, and Cyanobacteria (Figure 1). It showed the PHTs in plants form six monophyletic groups. Cluster I (PHT2 subfamily) is sister to the cyanobacteria and bacterial PHT proteins and constitutes a sister group to the Cluster II (PTB subfamily). Cluster III contained the grouping of the PHT4 family. Cluster IV contained the grouping of the PHT3 subfamily. The PHT1 subfamily only includes PHT genes in land plants and constitutes a sister group to the PTA subfamily. PHTs in land plants consisted of PHT1, PHT2, PHT3, and PHT4 subfamilies, while PHTs in algae contained PTA, PTB, PHT3, and PHT4 subfamilies. PHT1 proteins are the H^+^/Pi symporters that are responsible for Pi uptake in land plants, whereas PTB proteins are predicted to be the Na^+^/Pi symporters catalyzing Pi uptake in algae. Endosymbiotic theory posits that chloroplasts and mitochondria arose from bacteria. Both organelles have retained their PHTs in membranes. The PHT2, PHT3, and PHT4 gene families encode proteins that transport Pi between the cytoplasm and different subcellular compartments (chloroplasts and mitochondria) in land plants, while how Pi is transported in subcellular compartments in algae was still unclear. To further understand the PHT in algae, we chose a model alga Chlamydomonas to analyze the PHT gene family.

### Identification of *PHT* Gene Family in *Chlamydomonas reinhardtii*

To investigate the PHT genes in Chlamydomonas, we employed PHT protein sequences of Arabidopsis and rice in a global search against Chlamydomonas genomic databases. In all, 25 putative PHT genes were found in Chlamydomonas (Table 1). Details on gene name, locus name, open reading frame (ORF) length, protein length, molecular weight, isoelectric point (p*I*), predicted transmembrane domain (TM) number, predicted long hydrophilic loop (loop after), and P1BS element are listed in Table 1. The PTA subfamily contains 4 members, PTB contains 11 members, PHT3 contains 1 member, and PHT4 contains 9 members. We conducted blast analysis of the genes against the Pfam database and found that all proteins contain the MFS domain. Values for p*I* ranged from 6.21 to 9.63. The protein lengths of 25 putative PHTs range from 351 to 1666 aa, and their molecular weights are 37.44 to 171.66 kDa; most of them are around 60 kDa. PHT proteins contain 5 to 15 TM, most of them range from 10 to 12 TM, and most of them contain a large hydrophilic loop between the seventh and eighth TMDs in the PTB family except CrPTBx. The P1BS *cis* element sequence (GNATATNC) was determined previously as the binding motif for PHR1, and this element is present in the promoter regions of P starvation-induced genes in various higher plants (Zhou et al., 2008; Wang et al., 2014). To identify putative PSR1-binding sites, we searched the GNATATNC sequence in genome sequences of all CrPHT genes (Table 1). P1BS elements were identified in most of the CrPHT genes. P1BS elements were present in the promoter regions of CrPTA1 and CrPTA3. All PTB subfamilies except CrPTB2, CrPTB9, and CrPTB12 have P1BS, while there is no P1BS in the PHT3 family. In the PTB4 family, only CrPHT4-2 and CrPHT4-4 have P1BS elements.

**TABLE 1:** Key features of all 25 PHTs in *Chlamydomonas reinhardtii*.

Locus	Gene name	Gene length (bp)	ORF length (bp)	Protein length (aa)	Mol wt (k Da)	pI	TM number	Hydrophilic loop after	P1BS locations on PHT gene
Cre02.g075050	PTA1	5518	1683	560	62.57	8.72	11	–	55, 1760, 1771, 2488
Cre16.g686800	PTA2	5067	1719	572	63.63	9.2	11	–	–
Cre16.g686750	PTA3	5450	1722	573	63.39	8.91	11	–	329
Cre16.g686850	PTA4	6985	1671	556	61.93	8.97	11	–	–
Cre12.g491600	PTB1	11369	5001	1666	171.66	6.77	15	7	6097, 10563
Cre07.g325741	PTB2	5012	1959	652	70.11	8.51	12	7	–
Cre07.g325740	PTB3	5542	1959	652	70.19	8.65	12	7	190, 2344
Cre02.g144750	PTB4	4957	1929	642	69.13	8.22	11	7	1026, 2506
Cre02.g144700	PTB5	4578	1878	625	67.05	9.18	11	7	2169
Cre16.g655200	PTB6	6690	1839	612	65.12	8.71	11	7	287, 3502
Cre12.g489400	PTB7	9082	2508	835	85.43	8.02	13	7	512, 7432, 7445, 8258
Cre16.g676757	PTB8	9198	4020	1339	139.34	6.21	12	7	2439, 8766
Cre02.g144600	PTB9	4542	1887	628	67.04	8.63	11	7	–
Cre02.g144650	PTB12	6218	1875	624	66.76	8.63	11	7	–
Cre12.g493404	PTBx	14786	3879	1292	131.42	6.6	8	3	5218
Cre03.g172300	PHT3-1	2782	1056	351	37.44	9.17	5	–	–
Cre01.g023000	PHT4-1	6591	1644	547	54.17	9.63	12	–	–
Cre03.g187050	PHT4-3	4012	1857	618	61.83	9.83	13	–	–
Cre08.g379550	PHT4-4	5842	1725	574	60.59	9.27	11	–	25, 4928
Cre09.g396950	PHT4-9	6056	2193	730	76.08	9.52	12	–	–
Cre09.g399141	PHT4-2	6414	1305	434	46.64	9.75	11	–	1353
Cre12.g515750	PHT4-5	4476	1500	499	52.81	9.13	10	–	–
Cre16.g648300	PHT4-6	6153	1926	641	65.25	7.59	11	–	–
Cre16.g663600	PHT4-7	5604	1413	470	49.53	8.66	11	–	–
Cre16.g675300	PHT4-8	6254	1740	579	60.17	9.06	11	–	–

*The total number of predicted transmembrane domains (TM number) and the behind long hydrophilic loops (loop after) using a complete protein sequence. P1BS number in the genome regions of PT genes was identified by the NewPLACE database (http://sogo.dna.affrc.go.jp/cgi-bin).*

### Prediction of Subcellular Targeting of PHT Proteins in Chlamydomonas

An N-terminal signal peptide targets the protein to the secretory pathway; proteins are inserted into the endoplasmic reticulum (ER), and either remain in the ER or move to the plasma membrane, vacuole, or endomembrane compartments (Chrispeels and Raikhel, 1992). Using four online tools (details in section “Materials and Methods”), which could predict subcellular localization by detecting subcellular targeting motifs, we discovered the signal peptide at the N-terminus of all PHT proteins (Table 2). Among them, the entire PTA family was predicted to locate in the plasma membrane with a very high reliability class. PTB proteins were predicted to target to the secretory pathway. However, based on Bonnot’s report (Bonnot et al., 2017), PTB proteins are plasma membrane proteins, which suggests that they finally move to the plasma membrane through the secretory pathway. PHT3 was predicted to target to the mitochondrion, with a very low reliability class. Most of the PHT4 family proteins were localized in chloroplast with a reliability class between 4 and 5. CrPHT4-2 and CrPHT4-7 were predicted to target to the secretory pathway. The CrPHT4-5 has no signal peptide and did not display predicted localization.

**TABLE 2:** Prediction of subcellular targeting of PHT proteins in *Chlamydomonas reinhardtii*.

Locus	Name	PM	cTP	mTP	SP	Other	loc	RC
Cre02.g075050	PTA1	0.63	0.08	0.01	0.22	0.06	P	1
Cre16.g686800	PTA2	0.43	0.2	0.03	0.27	0.07	P	1
Cre16.g686750	PTA3	0.48	0.09	0.02	0.35	0.07	P	1
Cre16.g686850	PTA4	0.55	0.06	0.01	0.31	0.07	P	1
Cre12.g491600	PTB1	–	0.008	0.019	0.953	0.06	S	1
Cre07.g325741	PTB2	–	0.004	0.008	0.913	0.395	S	3
Cre07.g325740	PTB3	–	0.004	0.007	0.894	0.475	S	3
Cre02.g144750	PTB4	–	0.002	0.012	0.979	0.309	S	3
Cre02.g144700	PTB5	–	0.002	0.013	0.982	0.28	S	2
Cre16.g655200	PTB6	–	0.011	0.015	0.943	0.339	S	2
Cre12.g489400	PTB7	–	0.139	0.22	0.075	0.513	–	–
Cre16.g676757	PTB8	–	0.011	0.029	0.688	0.108	S	3
Cre02.g144600	PTB9	–	0.008	0.013	0.958	0.264	S	2
Cre02.g144650	PTB12	–	0.008	0.014	0.957	0.243	S	2
Cre12.g493404	PTBx	–	0.003	0.043	0.914	0.076	S	1
Cre03.g172300	PHT3-1	0.12	0.23	0.54	0.02	0.08	M	4
Cre01.g023000	PHT4-1	0.04	0.88	0.05	0.02	0.01	C	4
Cre03.g187050	PHT4-3	0.11	0.76	0.07	0.05	0.01	C	4
Cre08.g379550	PHT4-4	0.06	0.84	0.08	0.01	0.01	C	5
Cre09.g396950	PHT4-9	0.04	0.9	0.05	0.01	0	C	4
Cre09.g399141	PHT4-2	0.13	0.01	0.01	0.85	0.01	S	5
Cre12.g515750	PHT4-5	0.17	0.35	0.1	0.34	0.03	–	–
Cre16.g648300	PHT4-6	0.05	0.89	0.03	0.02	0.01	C	5
Cre16.g663600	PHT4-7	0.03	0	0	0.97	0	S	1
Cre16.g675300	PHT4-8	0.11	0.69	0.11	0.07	0.02	C	4

*PM, plasma membrane; cTP, chloroplast; mTP, mitochondrion; and SP, secretory pathway. The prediction was given with a reliability class (RC) indicated from 1 to 5. 1: 1.000 > diff > 0.800; 2: 0.800 > diff > 0.600; 3: 0.600 > diff > 0.400; 4: 0.400 > diff > 0.200; and 5: 0.200 > diff > 0.000. “–” means no data.*

### Conserved Motif Analysis of Chlamydomonas PHT Proteins

To analyze the conserved motifs and reveal the distinction between green algae and other ancient species, conserved motifs of PHT proteins from Chlamydomonas, bacteria, fungus, and metazoa were identified by MEME (Bailey and Elkan, 1994). The sequences of 4 Chlamydomonas PTA proteins; 11 PTB proteins; 8 PHT4 proteins; 5 PHT3 proteins; fungal PHT proteins ScPho89 and NcPho4; metazoan PHT proteins DmPit, HsPit1, and HsPit2; bacterial PHT proteins TePit, EcPitA, and EcPitB (from *Escherichia coli*); and 2 cyanobacteria proteins were selected for further analysis (Table 3). We identified a total of 35 conserved motifs among the selected PHT proteins and found that the conserved motifs could distinguish the Chlamydomonas PHT proteins from other members of the PHT proteins. Five motifs (motifs 1–5) were present in bacterial PHT, metazoan PHT, fungal PHT, and PTB family proteins. They contain the PHT signature GANDVAN domains (motif 2). Four motifs (motif 6–9) were common to the metazoan, fungal PHT, and PTB proteins. Twelve motifs (motifs 1–12) were present in PTB proteins. Eight motifs (motifs 16–23) were specific to the PTA proteins, and five motifs (motifs 24–28) were specific to the PHT3 proteins. Motifs 2, 4, and 29–35 were present in all PHT4 proteins. All motifs identified in the bacterial, metazoan, and fungal PHT proteins were present in the PTB proteins (Table 3). Meanwhile, motifs specific to the PTA and PHT3 family were found. Together, the conserved and specific motifs found in the different subfamilies of CrPHT proteins suggest their conservation in evolution and their divergence of functions.

**TABLE 3:** Overall conserved motifs of PHT families in Chlamydomonas, bacteria, metazoans, and fungi.

Motif	AA	Sequence	Bacterial PiT	Metazoan PiT	Fungal PiT	Chlamydomonas			
						
						PTA	PTB	PHT3	PHT4
Motif1	16	GLPvStTHcivGavvG	Y	Y	Y		Y		
Motif2	10	GANDVANafG	Y	Y	Y		Y		Y
Motif3	10	TPsRGFcaEL	Y	Y	Y		Y		
Motif4	6	sWfysp	Y	Y	Y		Y		Y
Motif5	16	TLrQAvljAavfEFsG	Y	Y	Y		Y		
Motif6	21	HaaAEVFdPetEyvykYLQVF		Y	Y		Y		
Motif7	16	PiWvLayGGaGlvyGL		Y	Y		Y		
Motif8	29	LRRebsttlaiwvlPllvllTvfLNvFFV		Y	Y		Y		
Motif9	10	VtrTiaggla		Y	Y		Y		
Motif10	13	KkAAtrGlnvDiH					Y		
Motif11	10	AWVaAciaaG					Y		
Motif12	13	aPSjtmeKdiakY					Y		
Motif13	9	GsGAVVWnD					Y		
Motif14	10	NtKSiGYvdP					Y		
Motif15	12	ADaEanaGKpKE					Y		
Motif16	18	DKPWMGRMRMQvMGFaWM				Y			
Motif17	26	WRKFGLLMyYYWHRNFGTAMSWFVWD				Y			
Motif18	22	DVTGLDLREGDKRWLAiLDGHH				Y			
Motif19	24	FQFLYYFSSFWGQFGPNATTWLLP				Y			
Motif20	23	PGLGMFCEAYFVFAVGNLSAlWK				Y			
Motif21	17	FGQLFLGFFADRiGRKW				Y			
Motif22	18	KRRGETVVLVFSMQGWGN				Y			
Motif23	30	GNKLFQGTFiKiiNPsASLIQVLEWTLLNS				Y			
Motif24	19	VKvnvQtnPgfykgjsdGf						Y	
Motif25	25	CKFGFYEyFKKtYsDmAGpEyakKY						Y	
Motif26	30	WGRQIPYTMMKFasFEttVEmiYKyavPkP						Y	
Motif27	30	SFagGYiAGVFCAIVSHPADNLVSFLNnaK						Y	
Motif28	28	MIGTLTGaQWGiYDAFKVfvGLPTTGGV						Y	
Motif29	22	DjapryAgallGltNTaGalaG							Y
Motif30	21	CNmDrvnmSVAijPMaaeFGW							Y
Motif31	10	GLVqSAFywG							Y
Motif32	14	WgyytlLsWLPTYF							Y
Motif33	12	AGvLLGjsNTAG							Y
Motif34	11	RvlvGlGzGVA							Y
Motif35	7	tVRKilQ							Y

*For each motif, the amino acid length (AA), the sequence, and the presence/absence (Y/–) in proteins of the PHT families (bacterial PiT, metazoan PiT, fungal PiT, PTA, PTB, PHT3, and PHT4) are given. Uppercase and lowercase indicate residues of the motifs conserved in >50% or <50%, respectively, of the concerned proteins.*

### Chromosomal Localization and Gene Duplication of *CrPHT* Genes

**FIGURE 2:**
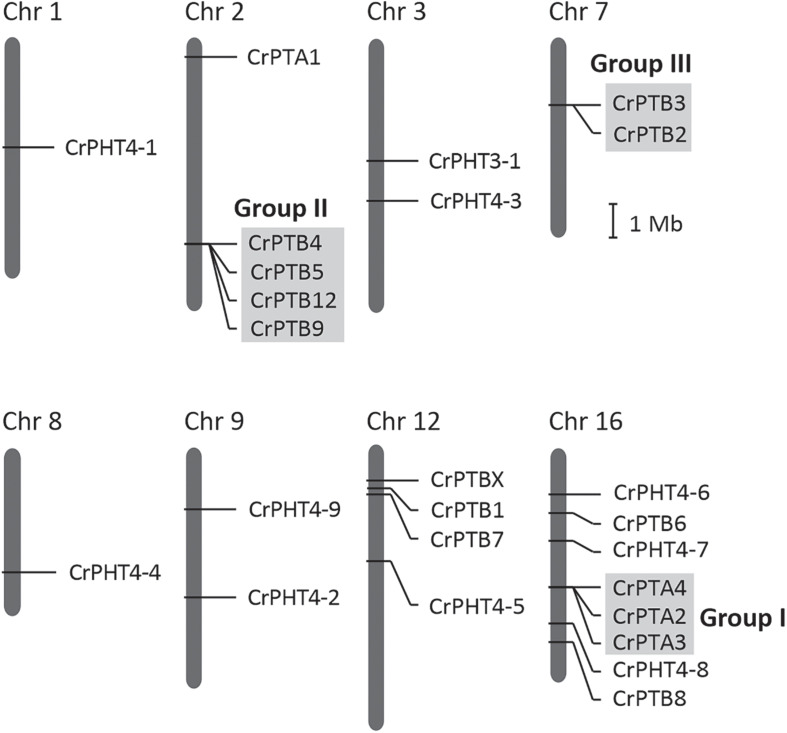
Chromosomal location and gene duplication of PHT proteins in Chlamydomonas. Chromosome numbers are shown at the top of each bar. Recent tandem duplicated genes were marked with a gray background. The scale presented on the left indicates chromosome sizes in megabases (Mb).

The chromosomal distributions of *CrPHT* genes were determined. As shown in Figure 2,25 PHT genes are distributed on 8 Chlamydomonas chromosomes. Out of the 25 predicted *PHT* genes, 18 are distributed across the 3 Chlamydomonas chromosomes (chromosome 2, 12 and 16) (Figure 2). Eight *PHT* genes scattered in clusters in chromosome 16. Chromosomes 1 and 8 each contain one gene. Chromosome 3, 7, and 9 each contain two genes. We further performed chromosome mapping to determine the gene duplication of *PHT* genes on the Chlamydomonas chromosomes. As shown in Supplementary Table 1, gene duplication of *CrPHT* genes contained four types, including tandem duplication (TD), proximal duplication (PD), transposed duplication (TRD), and dispersed duplication (DSD). Almost all the *PHT* genes were identified as duplicated genes (Supplementary Table 1). As shown in Figure 2, three groups of *PHT* genes can be identified as TD genes including group I (CrPTA2, CrPTA3, and CrPTA4) located on chromosome 16, group II (CrPTB4, CrPTB5, CrPTB9, and CrPTB12) located on chromosome 2, and group III (CrPTB2 and CrPTB3) located on chromosome 7, implying that the high density of PHT genes on this chromosome was mainly due to the TD events.

### Expression Profiles of *CrPHT* Genes During Pi Starvation

**FIGURE 3:**
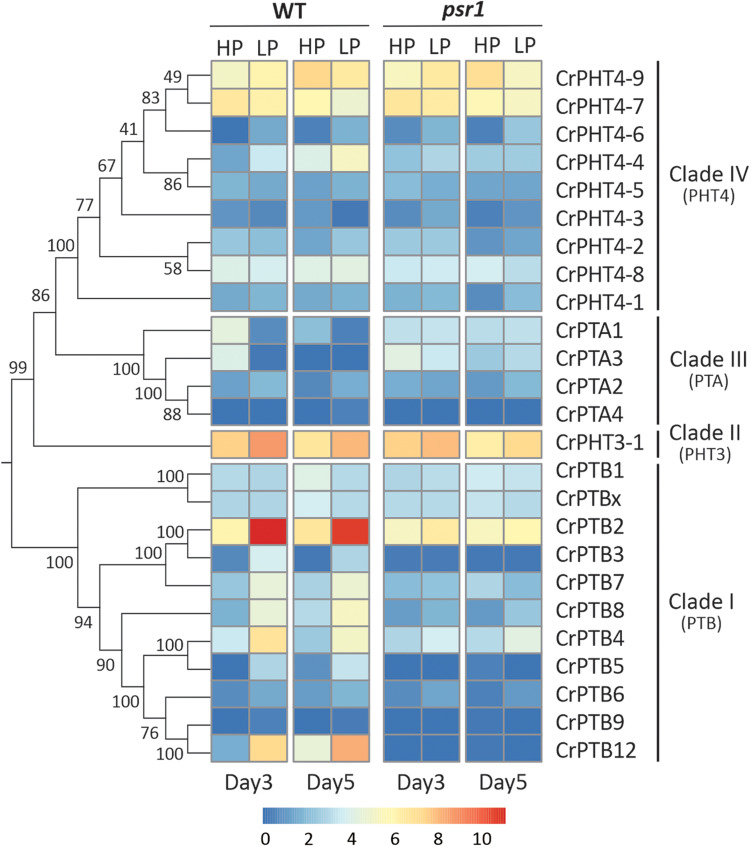
Expression profiles of Chlamydomonas PHT genes under phosphate-replete and starvation conditions. The phylogenetic tree of PHT proteins, showing in the left panel, was constructed by the neighbor-joining method with 1000 bootstrap replications using MEGA 7.0 software. Bootstrap values were shown on the branches. Heat map showing the expression of phosphate transporter genes under high-P (HP) and low-P (LP) conditions in day 3 and day 5 which display change in expression relative to WT HP conditions or *psr1* HP. Each value corresponds to log2 of normalized counts. Day 3 and day 5 mean equivalence to the onset of P starvation and 48 h after the onset of P starvation, respectively.

To uncover the expression profiles of *CrPHT* genes under deprivation starvation and whether they are controlled by CrPSR1, we reanalyzed the RNA-seq data from the database^1^ (Bajhaiya et al., 2016). Phylogenetic trees constructed by the NJ method showed that these CrPHT proteins could be divided into four clades (Figure 3). Transcription levels of *CrPHT* genes varied under Pi starvation (Figure 3 and Supplementary Table 2). *CrPTA1* and *CrPTA3* transcripts declined following exposure of nutrient-replete cells to P starvation, and this decline is less significant in the *psr1* mutant. This characteristic makes CrPTA1 and CrPTA3 candidates for the low-affinity Pi uptake system that operates in cells that are P-replete. The abundance of CrPTA2 and CrPTA4 transcripts is not significantly affected by P starvation. Upon Pi starvation, expressions of *CrPTB2*, *CrPTB3*, *CrPTB4*, *CrPTB5*, *CrPTB7*, *CrPTB8*, and *CrPTB12* were induced, and this induction does not occur in the *psr1* mutant, indicating that these genes were regulated by PSR1. The abundance of *CrPTB1*, *CrPTB9*, *CrPTB12*, and *CrPTBx* transcripts were not significantly affected by P starvation. The expression levels of genes in the *CrPHT3* and *CrPHT4* subfamilies were not significantly affected by P starvation, except *CrPHT4-3*, *CrPHT4-4*, and *CrPHT4-6*.

Noticeably, the expression profiles of genes in each TD group (Figure 2) are variable. In Group I, *CrPTA2* and *CrPTA4* are poorly expressed, while *CrPTA3* is highly expressed in nutrient-replete cells and its expression responded to phosphate starvation. This indicates that *CrPTA2* and *CrPTA4* are likely degenerated or redundant in function after duplication, compared to *CrPTA3*. In another duplicated group (Group III), the expression level of *CrPTB2* is much higher than *CrPTB3* under all the conditions, suggesting that the regulation of *CrPTB2* and *CrPTB3* are different. In Group III, *CrPTB4*, *CrPTB5*, and *CrPTB12* were sharply up-regulated under phosphate starvation, while CrPTB9 is barely expressed under phosphate starvation in both WT and *psr1* mutant. Different from other genes in Group III, *CrPTB4* still showed some expression in the *psr1* mutant. Together, functions of duplicated genes in each group are divergent and the gene duplication and subsequent divergence of *CrPHT* genes make up the complex network for P transport to adapt to a variable Pi environment.

### qRT-PCR Analyses of *CrPHT* Genes Under P Stress Conditions

**FIGURE 4:**
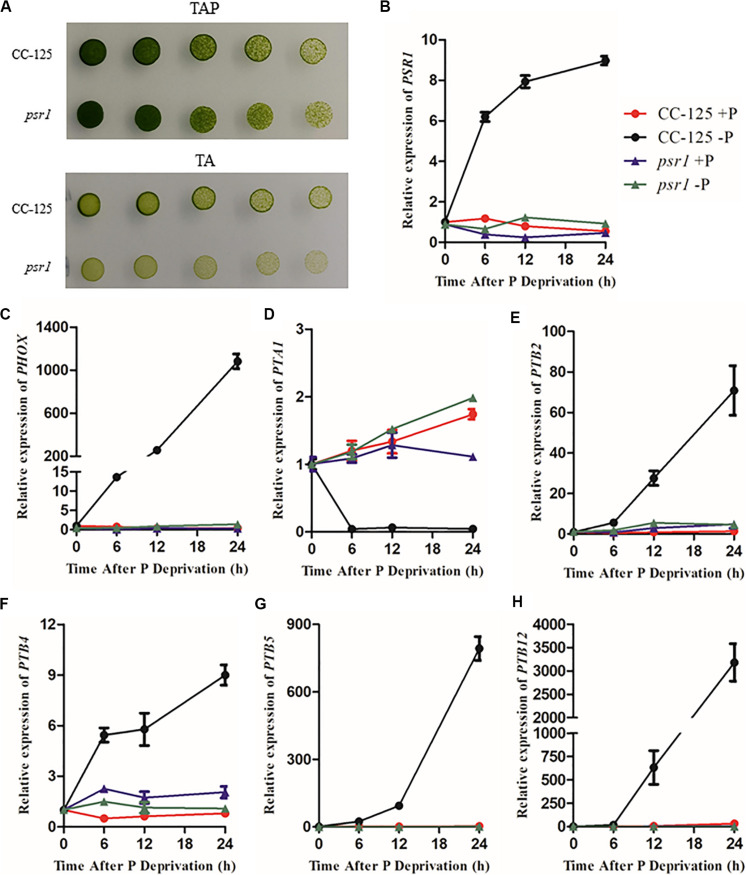
The relative expression of Chlamydomonas PHT genes under phosphate replete and starvation conditions. **(A)** Growth of CC-125 and *psr1* in the TAP or TA plate. **(B)** to **(H)**, The *CrPSR1* **(B)**, *CrPHOX* **(C)**, *CrPTA1* **(D)**, *CrPTB2* **(E)**, *CrPTB4* **(F)**, *CrPTB5* **(G)**, and *CrPTB12* **(H)** mRNA levels were quantified by qRT-PCR over a period of 0, 6, 12, and 24 h of phosphate deprivation. *CBLP* was used as the internal control. For **B–H**, data are the means ± s.d. All experiments were repeated three times, and similar results were obtained.

To explore the expression profiles of CrPHT genes under deprivation starvation, qRT-PCR analysis was performed (Figure 4). We firstly investigated the growth of WT and *psr1* mutant under P-replete or depleted conditions. In P-replete conditions, the growth of the *psr1* mutant is similar to that of WT. However, the *psr1* mutant was unable to acclimate properly to P starvation and exhibited much less growth than wild-type strains on the TA medium (Shimogawara et al., 1999). The *psr1* mutant showed growth defect compared to the WT in P-depleted conditions (Figure 4A). We further analyzed the steady-state transcript levels of genes encoding the phosphatase CrPHOX; the PHTs CrPTB2, CrPTB4, CrPTB5 CrPTB12, and CrPTA1; and the transcription factor CrPSR1 in the WT and *psr1* mutant (Figure 4). Consistent with previous report (Shimogawara et al., 1999), the expression of *CrPSR1* was significantly induced by P starvation (Figure 4B). *CrPHOX* was induced by P starvation in WT and not in the *psr1* mutant (Figure 4C). Under Pi starvation conditions, the expressions of *CrPTB2*, *CrPTB4*, *CrPTB5*, and *CrPTB12* were induced in the WT while they showed no effect in the *psr1* mutant. The *CrPTA1* transcript reduced following exposure of nutrient-replete cells to P starvation, and this decline does not occur in the *psr1* mutant. These results suggest that different *CrPHT* genes have different expression patterns and CrPSR1 displays an important role in regulating Pi starvation-dependent signaling in Chlamydomonas.

## Discussion

Phosphate is one of the most important nutrients that significantly affect plant growth and metabolism (Raghothama, 1999). Although the evolution and phylogenetic relationship of PHTs in different plant species have been reported, none of these studies addressed the possible origin and early evolution of plant PHTs. In this study, we identified members of the PHT family in Chlamydomonas and characterized their evolutionary and expression profiles under P starvation conditions. Interestingly, based on phylogenetic analyses of PHT family members among different plant species, we found that two Pi uptake systems exist in chlorophytes, including H^+^/Pi symport and Na^+^/Pi symport (Figure 1), while higher plants only contain a H^+^/Pi symport (Nussaume et al., 2011). PTB proteins are hypothesized to be Na^+^/Pi symporters, which facilitate Pi uptake in chlorophytes, whereas PHT1 proteins are H^+^/Pi symporters that facilitate Pi uptake in angiosperms and all streptophyte lineages (Ayadi et al., 2015; Bonnot et al., 2017). We suggest that the PTA subfamily in algae may also facilitate Pi uptake through H^+^/Pi symport.

In this study, 25 *CrPHT* genes were identified in the Chlamydomonas genome, comprising all four major types of plant PHT genes. Chromosome location analysis revealed that almost all the PHT genes were derived from multiple gene duplication events in Chlamydomonas. We found 4 *CrPTA* genes in Chlamydomonas, in which CrPTA1 and CrPTA3 are through gene duplication. All PTA proteins were predicted to be located in the plasma membrane, similar to the PHT1 in land plants. PHT1 proteins are the well-documented plant Pi transporters. These proteins have a conserved structure containing 12 transmembrane (TM) domains with a large hydrophilic loop between TM6 and TM7 and both hydrophilic N and C termini located in the cytoplasm (Raghothama, 1999; Nussaume et al., 2011). All plant PHT1 proteins have the conserved PHT1 signature GGDYPLSATIxSE and in fungi it is GGDYPLSxxIxSE (Karandashov and Bucher, 2005). In angiosperms, proteins in the PHT1 family catalyze the uptake of Pi from the environment through H^+^/Pi symport (Ayadi et al., 2015). We propose that the origin of PHT1 genes in land plants is worth more studies, to uncover the mechanism of PHT type bias. All PTB genes were located in chromosomes 2, 7, 12, and 16 and formed 4 clusters. We found a signal peptide in PTB proteins, and all PTB were predicted to the secretory pathway. For example, MpPTB proteins traffic to the plasma membrane through the secretory pathway and function as the plasma membrane PHT for Pi uptake (Bonnot et al., 2017). It indicated that CrPTB may also locate on the plasma membrane and control the Pi uptake in Chlamydomonas.

The topology of the phylogenetic tree showed that the PHT2 subfamily is sister to the bacterial PHT proteins, together with a sister group to the PTB subfamily. We inferred that the PTB protein may move and locate to the chloroplast during evolution because we did not find PHT2 in chlorophyte algae, while in land plants, such as rice, PHT2;1 localized at the chloroplast envelope and functioned as a low-affinity Pi transporter (Liu et al., 2019). PHT3 proteins have two TM α-helices separated by a hydrophilic extramembrane loop, which are conserved in all analyzed mitochondrial transporter proteins, being essential for mitochondrial targeting (Zhu et al., 2012). There are three members in Arabidopsis, as well as in a basic angiosperm, *Amborella trichopoda*, while six transporters in rice and nine in *Physcomitrella patens* were clustered with AtPHT3 proteins in the phylogenetic tree (Wang et al., 2017). We only found one PHT3 gene in Chlamydomonas. Therefore, PHT3 family rapidly expanded in land plants, especially in mosses and monocots, through gene duplication during the evolution of plant lineages. In contrast to the rapid expansion of PHT3 in land plants, the members of the PHT4 family are ancient and relatively consistent. The PHT4 family was first characterized and designated by Guo et al. (2008) in Arabidopsis (Guo et al., 2008). There are six members in the PHT4 family in Arabidopsis and seven putative PHT4 members in rice (Wang et al., 2017). PHT4 proteins share a similarity with SLC17/type I Pi transporters. We found nine PHT4 genes in Chlamydomonas which contained 11–13 TM and mainly located in the chloroplast.

Transcription of *CrPHT* genes varies in response to changes of phosphate levels. We found that, upon Pi starvation, expression of *CrPTA1* and *CrPTA3* is reduced in the cells. This characteristic makes CrPTA1 and CrPTA3 candidates for the low-affinity Pi uptake system that operates in cells under P-replete conditions. Most Arabidopsis PHT1 family genes, which encode H^+^/Pi symporters, are up-regulated in phosphate-deprived conditions (Mudge et al., 2002). We also found that P starvation led to enhanced expression levels of *CrPTB2*, *CrPTB3*, *CrPTB4*, *CrPTB5*, *CrPTB6*, *CrPTB7*, and *CrPTB8*, indicating that they may be high-affinity PHTs operating at low Pi conditions. We also found that the reduction of *CrPTA* and induction of *CrPTB* transcription does not occur in *psr1* mutant (Figure 4), consistent with that the P1BS element is present in these genes (Table 1). Our results suggest that they may be regulated by CrPSR1.

## Materials and Methods

### Sequence Collection and Phylogenetic Analyses

To illuminate the evolutionary relationship of PHT gene families in plant lineages, the representative PHT genes from *Arabidopsis thaliana* (At), *Oryza sativa* (Os), *Amborella trichopoda* (Atr), and *Volvox carteri* (Vc). *Physcomitrella patens* (Pp), *Chlamydomonas reinhardtii* (Cr), and *Cyanidioschyzon merolae* (Cm) were selected for constructing a phylogenetic tree with PHT genes from *Escherichia coli* (Ec) and Cyanobacteria as outgroup. Multi-sequence alignments were performed by Clustal Omega (Sievers et al., 2011), and a phylogenetic tree was reconstructed by IQTREE using the maximum likelihood method (Nguyen et al., 2015). WAG + F + R6 was the best-fitting evolutionary model selected by ModelFinder under Bayesian Information Criterion (Kalyaanamoorthy et al., 2017). The gene name referred to Liu’s report (Liu et al., 2011).

### Sequence Analysis and Structural Characterization

Information of candidate PHT genes in Chlamydomonas was obtained via searching the Phytozome database^2^ (Goodstein et al., 2012), including chromosome locations, genomic sequences, coding sequences (CDS), and amino acid sequences. Conserved motifs were identified by MEME (Bailey and Elkan, 1994). Molecular weight, theoretical isoelectric point (theoretical p*I*), and instability index (II; with the value > 40 classified as unstable) of CrPHT proteins were analyzed by the ProtParam tool^3^. Hydropathy profiles were predicted using TMPRED^4^. P1BS was identified by the NewPLACE database^5^ (Higo et al., 1999).

### Subcellular Localization and Conserved Motif Analysis of CrPHT

Four Online tools were employed to predict subcellular localization, including Wolf TargetP^6^ (Emanuelsson et al., 2007), Psort^7^, MultiLoc^8^, and YLoc^9^. The prediction of the signal peptide and TM was performed with the SMART program^10^. The putative protein sequences were subjected to MEME program^11^ to investigate conserved motifs with the following parameters: site distribution—any number of repetitions, number of motifs—35, the motif width between 5 and 30 (Bailey and Elkan, 1994).

### Chromosome Localization and Gene Duplication

Chromosome mapping of the candidate CrPHT genes was viewed using the software MapDraw V2.1 (Yu et al., 2016). Gene duplication was identified based on plantDGD^12^ (Qiao et al., 2019).

### Expression Profile of CrPHT Genes Under P Stress Conditions

To get an insight into the expression profiles of the CrPHT gene family under P deprivation, we reanalyzed the RNA-seq sequence data in the ArrayExpress database^13^ under the accession number E-MTAB-2556 (Bajhaiya et al., 2016). In detail, wild-type (CC125) and *psr1* mutant cells were cultured until day 3 and day 5 in high-P and low-P TAP media. For the low-P wild-type cells, day 3 and day 5 were equivalent to onset of P starvation and 48 h post-P starvation, respectively. Normalized log2 values of expression levels are shown in Supplementary Table 2.

### Algae Growth Conditions, RNA Extraction, cDNA Synthesis, and Expression Analysis

The *C. reinhardtii* WT strain CC-125 and CC-4350 (*Crpsr1-1*) mutants were obtained from the Chlamydomonas Resource Center (Li et al., 2016). Cells were cultured in standard Tris–acetate-phosphate TAP medium at pH 7.0 under continuous illumination (50 mmol photons m^–2^ s^–1^) on a rotating platform (150 rpm) at 24°C. For Pi deprivation, cells in the mid-logarithmic phase (5–8 × 10^6^ cells mL^–1^) were pelleted by centrifugation (2,000 *g*, 5 min), washed twice with TA in which 1.5 mM potassium chloride which was substituted for 1 mM potassium phosphate (Quisel et al., 1996), and then resuspended in TA medium.

Total RNA was extracted from frozen cell pellets using the RNeasy Mini Kit (Qiagen) and reverse transcribed to complementary DNA after DNase I treatment following the manufacturer’s instructions (NEB). Quantitative real-time PCR was performed using a SYBR Premix kit (Roche) on a QuantStudio 6 Flex machine (Life Technologies). The *CBLP* gene was used as an internal control (Chang et al., 2005). The primer pairs used for RT-qPCR are given in the Supplementary Table 3.

## Data Availability Statement

The raw data supporting the conclusions of this article will be made available by the authors, without undue reservation.

## Author Contributions

YZ and HYZ conceived and supervised the project. LW, LX, YZ, and HYZ designed the research. LW and LX performed the experiments. LW, HY, LX, GC, and HZ analyzed the data. LW, HZ, and HYZ wrote the manuscript. All authors discussed the results and commented on the manuscript.

## Conflict of Interest

The authors declare that the research was conducted in the absence of any commercial or financial relationships that could be construed as a potential conflict of interest.

## Footnotes

1 http://www.ebi.ac.uk/arrayexpress

2 https://phytozome.jgi.doe.gov/pz/portal.html

3 http://web.expasy.org/protparam/

4 http://www.ch.embnet.org/software/TMPRED_form.html

5 http://sogo.dna.affrc.go.jp/cgi-bin/

6 http://www.cbs.dtu.dk/services/TargetP/

7 http://www.genscript.com/psort/wolf_psort.html

8 http://abi.inf.unituebingen.de/Services/MultiLoc2

9 https://abi-services.informatik.uni-tuebingen.de/yloc/webloc.cgi

10 http://smart.embl-heidelberg.de/smart/batch.pl

11 http://meme-suite.org/tools/meme

12 http://pdgd.njau.edu.cn:8080

13 http://www.ebi.ac.uk/arrayexpress

## Supplementary Material

Supplementary Table 1 List of gene duplication pairs identified in CrPHT genes.

Supplementary Table 2 Expression level of CrPHT genes under phosphate starvation in the WT and *psr1* mutant.

Supplementary Table 3 List of all primers used in this study.

## References

[B1] Ayadi A. David P. Arrighi J. F. Chiarenza S. Thibaud M. C. Nussaume L. (2015). Reducing the genetic redundancy of *Arabidopsis* PHOSPHATE TRANSPORTER1 transporters to study phosphate uptake and signaling. *Plant Physiol.* 167 1511–1526. 10.1104/pp.114.252338 25670816 PMC4378149

[B2] Bailey T. L. Elkan C. (1994). Fitting a mixture model by expectation maximization to discover motifs in biopolymers. *Proc. Int. Conf. Intell. Syst. Mol. Biol.* 2 28–36. 7584402

[B3] Bajhaiya A. K. Dean A. P. Zeef L. A. Webster R. E. Pittman J. K. (2016). PSR1 is a global transcriptional regulator of phosphorus deficiency responses and carbon storage metabolism in *Chlamydomonas reinhardtii*. *Plant Physiol.* 170 1216–1234. 10.1104/pp.15.01907 26704642 PMC4775146

[B4] Bonnot C. Proust H. Pinson B. Colbalchini F. P. L. Lesly-Veillard A. Breuninger H. (2017). Functional PTB phosphate transporters are present in streptophyte algae and early diverging land plants. *New Phytol.* 214 1158–1171. 10.1111/nph.14431 28134432

[B5] Bustos R. Castrillo G. Linhares F. Puga M. I. Rubio V. Perez-Perez J. (2010). A central regulatory system largely controls transcriptional activation and repression responses to phosphate starvation in *Arabidopsis*. *PLoS Genet.* 6:e001102. 10.1371/journal.pgen.1001102 20838596 PMC2936532

[B6] Chang C. W. Moseley J. L. Wykoff D. Grossman A. R. (2005). The LPB1 gene is important for acclimation of *Chlamydomonas reinhardtii* to phosphorus and sulfur deprivation. *Plant Physiol.* 138 319–329. 10.1104/pp.105.059550 15849300 PMC1104186

[B7] Chrispeels M. J. Raikhel N. V. (1992). Short peptide domains target proteins to plant vacuoles. *Cell* 68 613–616. 10.1016/0092-8674(92)90134-x 1739969

[B8] Emanuelsson O. Brunak S. Von Heijne G. Nielsen H. (2007). Locating proteins in the cell using TargetP, SignalP and related tools. *Nat. Protoc.* 2 953–971. 10.1038/nprot.2007.131 17446895

[B9] Goodstein D. M. Shu S. Howson R. Neupane R. Hayes R. D. Fazo J. (2012). Phytozome: a comparative platform for green plant genomics. *Nucleic Acids Res.* 40 D1178–D1186. 22110026 10.1093/nar/gkr944 PMC3245001

[B10] Guo B. Jin Y. Wussler C. Blancaflor E. B. Motes C. M. Versaw W. K. (2008). Functional analysis of the *Arabidopsis* PHT4 family of intracellular phosphate transporters. *New Phytol.* 177 889–898. 10.1111/j.1469-8137.2007.02331.x 18086223

[B11] Higo K. Ugawa Y. Iwamoto M. Korenaga T. (1999). Plant cis-acting regulatory DNA elements (PLACE) database: 1999. *Nucleic Acids Res.* 27 297–300. 10.1093/nar/27.1.297 9847208 PMC148163

[B12] Kalyaanamoorthy S. Minh B. Q. Wong T. K. F. Von Haeseler A. Jermiin L. S. (2017). ModelFinder: fast model selection for accurate phylogenetic estimates. *Nat. Methods* 14 587–589. 10.1038/nmeth.4285 28481363 PMC5453245

[B13] Karandashov V. Bucher M. (2005). Symbiotic phosphate transport in arbuscular mycorrhizas. *Trends Plant Sci.* 10 22–29. 10.1016/j.tplants.2004.12.003 15642520

[B14] Kobayashi I. Fujiwara S. Shimogawara K. Kaise T. Usuda H. Tsuzuki M. (2003). Insertional mutagenesis in a homologue of a Pi transporter gene confers arsenate resistance on *Chlamydomonas*. *Plant Cell Physiol.* 44 597–606. 10.1093/pcp/pcg081 12826625

[B15] Kumar Sharma A. Muhlroth A. Jouhet J. Marechal E. Alipanah L. Kissen R. (2020). The Myb-like transcription factor phosphorus starvation response (PtPSR) controls conditional P acquisition and remodelling in marine microalgae. *New Phytol.* 225 2380–2395. 10.1111/nph.16248 31598973

[B16] Li X. Zhang R. Patena W. Gang S. S. Blum S. R. Ivanova N. (2016). An indexed, mapped mutant library enables reverse genetics studies of biological processes in *Chlamydomonas reinhardtii*. *Plant Cell* 28 367–387. 10.1105/tpc.15.00465 26764374 PMC4790863

[B17] Liu F. Chang X.-J. Ye Y. Xie W.-B. Wu P. Lian X.-M. (2011). Comprehensive sequence and whole-life-cycle expression profile analysis of the phosphate transporter gene family in rice. *Mol. Plant.* 4 1105–1122. 10.1093/mp/ssr058 21832284

[B18] Liu X. L. Wang L. Wang X. W. Yan Y. Yang X. L. Xie M. Y. (2019). Mutation of the chloroplast-localized phosphate transporter OsPHT2;1 reduces flavonoids accumulation and UV tolerance in rice. *Plant J.* 102 53–67. 10.1111/tpj.14611 31733118

[B19] Lorenzo-Orts L. Couto D. Hothorn M. (2020). Identity and functions of inorganic and inositol polyphosphates in plants. *New Phytol.* 225 637–652. 10.1111/nph.16129 31423587 PMC6973038

[B20] Mimura T. Reid R. J. Ohsumi Y. Smith F. A. (2002). Induction of the Na+/Pi cotransport system in the plasma membrane of *Chara corallina* requires external Na+ and low levels of Pi. *Plant Cell Environ.* 25 1475–1481. 10.1046/j.1365-3040.2002.00921.x

[B21] Moseley J. L. Chang C. W. Grossman A. R. (2006). Genome-based approaches to understanding phosphorus deprivation responses and PSR1 control in *Chlamydomonas reinhardtii*. *Eukaryot. Cell* 5 26–44. 10.1128/ec.5.1.26-44.2006 16400166 PMC1360252

[B22] Moseley J. L. Gonzalez-Ballester D. Pootakham W. Bailey S. Grossman A. R. (2009). Genetic interactions between regulators of *Chlamydomonas* phosphorus and sulfur deprivation responses. *Genetics* 181 889–905. 10.1534/genetics.108.099382 19087952 PMC2651062

[B23] Mudge S. R. Rae A. L. Diatloff E. Smith F. W. (2002). Expression analysis suggests novel roles for members of the Pht1 family of phosphate transporters in *Arabidopsis*. *Plant J.* 31 341–353. 10.1046/j.1365-313x.2002.01356.x 12164813

[B24] Nguyen L. T. Schmidt H. A. Von Haeseler A. Minh B. Q. (2015). IQ-TREE: a fast and effective stochastic algorithm for estimating maximum-likelihood phylogenies. *Mol. Biol. Evol.* 32 268–274. 10.1093/molbev/msu300 25371430 PMC4271533

[B25] Nussaume L. Kanno S. Javot H. Marin E. Pochon N. Ayadi A. (2011). Phosphate import in plants: focus on the PHT1 transporters. *Front. Plant Sci.* 2:83. 10.3389/fpls.2011.00083 22645553 PMC3355772

[B26] Qiao X. Li Q. Yin H. Qi K. Li L. Wang R. (2019). Gene duplication and evolution in recurring polyploidization-diploidization cycles in plants. *Genome Biol.* 20:38. 10.1186/s13059-019-1650-2 PMC6383267 30791939

[B27] Quisel J. D. Wykoff D. D. Grossman A. R. (1996). Biochemical characterization of the extracellular phosphatases produced by phosphorus-deprived *Chlamydomonas reinhardtii*. *Plant Physiol.* 111 839–848. 10.1104/pp.111.3.839 8754684 PMC157902

[B28] Raghothama K. G. (1999). Phosphate acquisition. *Annu. Rev. Plant Physiol. Plant Mol. Biol.* 50 665–693. 15012223 10.1146/annurev.arplant.50.1.665

[B29] Reid R. J. Mimura T. Ohsumi Y. Walker N. A. Smith F. A. (2000). Phosphate uptake in chara: membrane transport via Na/Pi cotransport. *Plant Cell Environ.* 23 223–228. 10.1046/j.1365-3040.2000.00524.x

[B30] Shimogawara K. Wykoff D. D. Usuda H. Grossman A. R. (1999). *Chlamydomonas reinhardtii* mutants abnormal in their responses to phosphorus deprivation. *Plant Physiol.* 120 685–694. 10.1104/pp.120.3.685 10398703 PMC59306

[B31] Sievers F. Wilm A. Dineen D. Gibson T. J. Karplus K. Li W. Z. (2011). Fast, scalable generation of high-quality protein multiple sequence alignments using Clustal Omega. *Mol. Syst. Biol.* 7:539. 10.1038/msb.2011.75 21988835 PMC3261699

[B32] Sun L. Song L. Zhang Y. Zheng Z. Liu D. (2016). *Arabidopsis* PHL2 and PHR1 act redundantly as the key components of the central regulatory system controlling transcriptional responses to phosphate starvation. *Plant Physiol.* 170 499–514. 10.1104/pp.15.01336 26586833 PMC4704584

[B33] Wang D. Lv S. Jiang P. Li Y. (2017). Roles, regulation, and agricultural application of plant phosphate transporters. *Front. Plant Sci.* 8:817 10.3389/fpls.2011.00817 PMC5435767 28572810

[B34] Wang Z. Y. Ruan W. Y. Shi J. Zhang L. Xiang D. Yang C. (2014). Rice SPX1 and SPX2 inhibit phosphate starvation responses through interacting with PHR2 in a phosphate-dependent manner. *Proc. Natl. Acad. Sci. U.S.A.* 111 14953–14958. 10.1073/pnas.1404680111 25271318 PMC4205599

[B35] Wykoff D. D. Grossman A. R. Weeks D. P. Usuda H. Shimogawara K. (1999). Psr1, a nuclear localized protein that regulates phosphorus metabolism in *Chlamydomonas*. *Proc. Natl. Acad. Sci. U.S.A.* 96 15336–15341. 10.1073/pnas.96.26.15336 10611385 PMC24820

[B36] Yu J. Cheng Y. Feng K. Ruan M. Ye Q. Wang R. (2016). Genome-wide identification and expression profiling of tomato Hsp20 gene family in response to biotic and abiotic stresses. *Front. Plant Sci.* 7:1215 10.3389/fpls.2011.01215 PMC4987377 27582749

[B37] Zhou J. Jiao F. Wu Z. Li Y. Wang X. He X. (2008). OsPHR2 is involved in phosphate-starvation signaling and excessive phosphate accumulation in shoots of plants. *Plant Physiol.* 146 1673–1686. 10.1104/pp.107.111443 18263782 PMC2287342

[B38] Zhu W. Miao Q. Sun D. Yang G. D. Wu C. G. Huang J. G. (2012). The mitochondrial phosphate transporters modulate plant responses to salt stress via affecting ATP and gibberellin metabolism in *Arabidopsis thaliana*. *PLoS One* 7:e043530. 10.1371/journal.pone.0043530 22937061 PMC3427375

