# Supplementary material for: Genome-Wide Identification, Expression Profiling, and Evolution of Phosphate Transporter Gene Family in Green Algae
Source: Front Genet. 2020 Oct 8;11:590947. doi: 10.3389/fgene.2020.590947 (PMC7578391; doi:10.3389/fgene.2020.590947)
Supplement: Supplementary Table 1 — List of gene duplication pairs identified in CrPHT genes. [file Data_Sheet_1.docx]

Supplementary Material

**Table S1. List of gene duplication pairs identified in CrPHT genes.**

| Duplicate 1 | PTA1 | PTA2 | PTA2 | PTA3 | PTB1 | PTB4 | PTB4 | PTB3 | PTB4 |
| --- | --- | --- | --- | --- | --- | --- | --- | --- | --- |
| Duplicate 2 | PTA3 | PTA4 | PTA3 | PTA4 | PTBx | PTB12 | PTB6 | PTB4 | PTB8 |
| E-value | 0 | 0 | 0 | 0 | 1E-99 | 0 | 0 | 2E-129 | 3E-58 |
| Type | DSD | TD | PD | DSD | DSD | DSD | DSD | DSD | DSD |
| Duplicate 1 | PTB5 | PTB5 | PTB3 | PTB8 | PTB2 | PTB2 | PTB9 | PTB5 | PTB4 |
| Duplicate 2 | PTB9 | PTB6 | PTB7 | PTBX | PTB6 | PTB3 | PTB12 | PTB9 | PTB5 |
| E-value | 0 | 0 | 8E-86 | 4E-66 | 8E-123 | 0 | 0 | 0 | 0 |
| Type | DSD | DSD | DSD | DSD | DSD | TD | TD | TD | PD |
| Duplicate 1 | PHT4-1 | PHT4-3 | PHT4-4 | PHT4-2 | PHT4-4 | PHT4-7 | PHT4-5 | PHT4-7 | PHT4-6 |
| Duplicate 2 | PHT4-4 | PHT4-4 | PHT4-7 | PHT4-4 | PHT4-6 | PHT4-9 | PHT4-7 | PHT4-8 | PHT4-9 |
| E-value | 2E-38 | 4E-35 | 3E-89 | 6E-69 | 1E-63 | 8E-83 | 2E-43 | 2E-44 | 3E-88 |
| Type | DSD | DSD | DSD | DSD | DSD | DSD | DSD | DSD | TRD |

Gene duplication data was reanalyzed from Plant duplicate gene database (http://pdgd.njau.edu.cn:8080). Four types of duplication events are determined, tandem duplication (TD), proximal duplication (PD), transposed duplication (TRD) and dispersed duplication (DSD).

**Table S2. Expression level of CrPHT genes under phosphate starvation in the WT and *psr1* mutant.**

|  | Day3 | | | | Day5 | | | |
| --- | --- | --- | --- | --- | --- | --- | --- | --- |
|  | WT HP | WT LP | *psr1* HP | *psr1* LP | WT HP | WT LP | *psr1* HP | *psr1* LP |
| PTA1 | 4.434153 | 0.696297 | 3.268053 | 3.391193 | 2.159467 | 0.358877 | 3.214876 | 3.306789 |
| PTA2 | 1.267865 | 1.961595 | 1.614342 | 1.437508 | 0.616792 | 1.573857 | 1.03874 | 1.987395 |
| PTA3 | 4.095249 | 0.141943 | 4.327034 | 3.539883 | 0 | 0 | 2.541085 | 3.059179 |
| PTA4 | 0 | 0 | 0 | 0 | 0 | 0.358877 | 0 | 0 |
| PTB1 | 3.060389 | 2.948668 | 2.932848 | 3.205743 | 4.196725 | 3.092784 | 3.637857 | 3.457865 |
| PTB2 | 6.082826 | 11.22681 | 5.360178 | 6.441825 | 6.779077 | 10.80756 | 5.462089 | 6.035849 |
| PTB3 | 0.587725 | 3.892807 | 0.330805 | 0.250763 | 0.122885 | 3.027657 | 0.202428 | 0.20922 |
| PTB4 | 3.556003 | 6.867859 | 2.968911 | 3.797933 | 2.514057 | 5.22987 | 3.067128 | 4.335983 |
| PTB5 | 0 | 3.005495 | 0.090079 | 0 | 0.848151 | 3.456691 | 0.379916 | 0 |
| PTB6 | 0.681179 | 1.567746 | 0.717462 | 1.403403 | 1.16655 | 1.86235 | 0.379916 | 1.065189 |
| PTB7 | 2.439703 | 4.505379 | 2.099587 | 2.336238 | 2.816783 | 4.748956 | 2.959492 | 2.096711 |
| PTB8 | 1.771379 | 4.631089 | 1.234504 | 1.87094 | 3.097167 | 5.331185 | 1.03874 | 2.382311 |
| PTB9 | 0 | 0.389754 | 0 | 0 | 0 | 0.248881 | 0.202428 | 0 |
| PTB12 | 1.683764 | 7.321123 | 0.090079 | 0.08852 | 4.694594 | 8.321302 | 0.202428 | 0 |
| PTBx | 2.933186 | 2.986801 | 3.071987 | 3.081833 | 3.749259 | 3.076776 | 3.471504 | 3.138023 |
| PHT3-1 | 7.540388 | 8.791813 | 7.562266 | 8.049154 | 6.843043 | 8.112407 | 6.328832 | 7.329204 |
| PHT4-1 | 1.49075 | 1.882895 | 1.730938 | 1.943938 | 1.474153 | 1.704617 | 0.680354 | 2.043088 |
| PHT4-2 | 2.412707 | 2.174811 | 2.511797 | 2.490403 | 1.427228 | 2.388581 | 0.928882 | 1.356501 |
| PHT4-3 | 0.929923 | 0.601174 | 0.717462 | 1.470826 | 1.047484 | 0.1298 | 0.379916 | 0.953362 |
| PHT4-4 | 1.383607 | 3.516319 | 2.338856 | 3.015644 | 4.057294 | 5.283162 | 2.648964 | 2.656761 |
| PHT4-5 | 1.893566 | 1.567746 | 2.033011 | 1.626766 | 1.276534 | 1.785638 | 1.40978 | 1.356501 |
| PHT4-6 | 0 | 1.516531 | 0.717462 | 1.845763 | 0.34111 | 1.785638 | 0.379916 | 2.466193 |
| PHT4-7 | 6.690484 | 6.24909 | 6.745607 | 6.425986 | 6.01281 | 4.90216 | 5.822314 | 5.305521 |
| PHT4-8 | 4.137083 | 3.820746 | 3.499349 | 3.688898 | 4.265032 | 4.295741 | 3.706051 | 3.236864 |
| PHT4-9 | 5.229154 | 6.122074 | 5.437284 | 6.529424 | 7.500899 | 6.621138 | 7.170943 | 5.3225 |

Log2 values of normalized expression levels were shown in the table. That day 3 and day 5 means equivalent to the onset of P starvation, and 48 h after the onset of P starvation, respectively.

**Table S3** List of all primers used in this study.

| Primer names | | | Sequence (5’-3’) Use of primers | | |
| --- | --- | --- | --- | --- | --- |
| CBLP.QF | CTTCTCGCCCATGACCAC | | Quantitative RT-PCR |  |  |
| CBLP.QR | CCCACCAGGTTGTTCTTCAG | | Quantitative RT-PCR |  |  |
| PSR1.QF | ACAGCAGCAACAAGAGCAAC | | Quantitative RT-PCR |  |  |
| PSR1.QR | CGAAATCACCGAAGTCAAAG | | Quantitative RT-PCR |  |  |
| PHOX.QF | TTCCGTTTCCGTTCTCTGAC | | Quantitative RT-PCR |  |  |
| PHOX.QR | CCCTGCATCTTGTTCTCCAG | | Quantitative RT-PCR |  |  |
| PTA1.QF | TGGGCGCATTCATGTTACCT | | Quantitative RT-PCR |  |  |
| PTA1.QR | CAAAGAAGCCCAACACCAGC | | Quantitative RT-PCR |  |  |
| PTB5.QF | CTCAACCCAGTTGGCAATTTACTTT | | Quantitative RT-PCR |  |  |
| PTB5.QR | GCCTTGTTCGAGTCCCAGT | | Quantitative RT-PCR |  |  |
| PTB4.QF | CCAACCTGGCAATCTACATG | | Quantitative RT-PCR |  |  |
| PTB4.QR | GCCTTGTTCGAGTCCCAGT | | Quantitative RT-PCR |  |  |
| PTB2.QF | AGACGGCTGAACAGTGCTAC | | Quantitative RT-PCR |  |  |
| PTB2.QR | CGTGGAGACCCATATGACCG | | Quantitative RT-PCR |  |  |
| PTB12.QF | TTGTCGGACATGTGGACTGG | | Quantitative RT-PCR |  |  |
| PTB12.QR | CAGCCCCGCCTATCGTATTT | | Quantitative RT-PCR |  |  |
